# Supplementary material for: Restriction of Zika Virus Replication in Human Monocyte-Derived Macrophages by Pro-Inflammatory (M1) Polarization
Source: Int J Mol Sci. 2025 Jan 23;26(3):951. doi: 10.3390/ijms26030951 (PMC11816608; doi:10.3390/ijms26030951)
Supplement: Supplementary file 1 [file ijms-26-00951-s001.zip › ijms-3307112-supplementary.pdf]

Supplemental Figures

Supplemental Figure S1

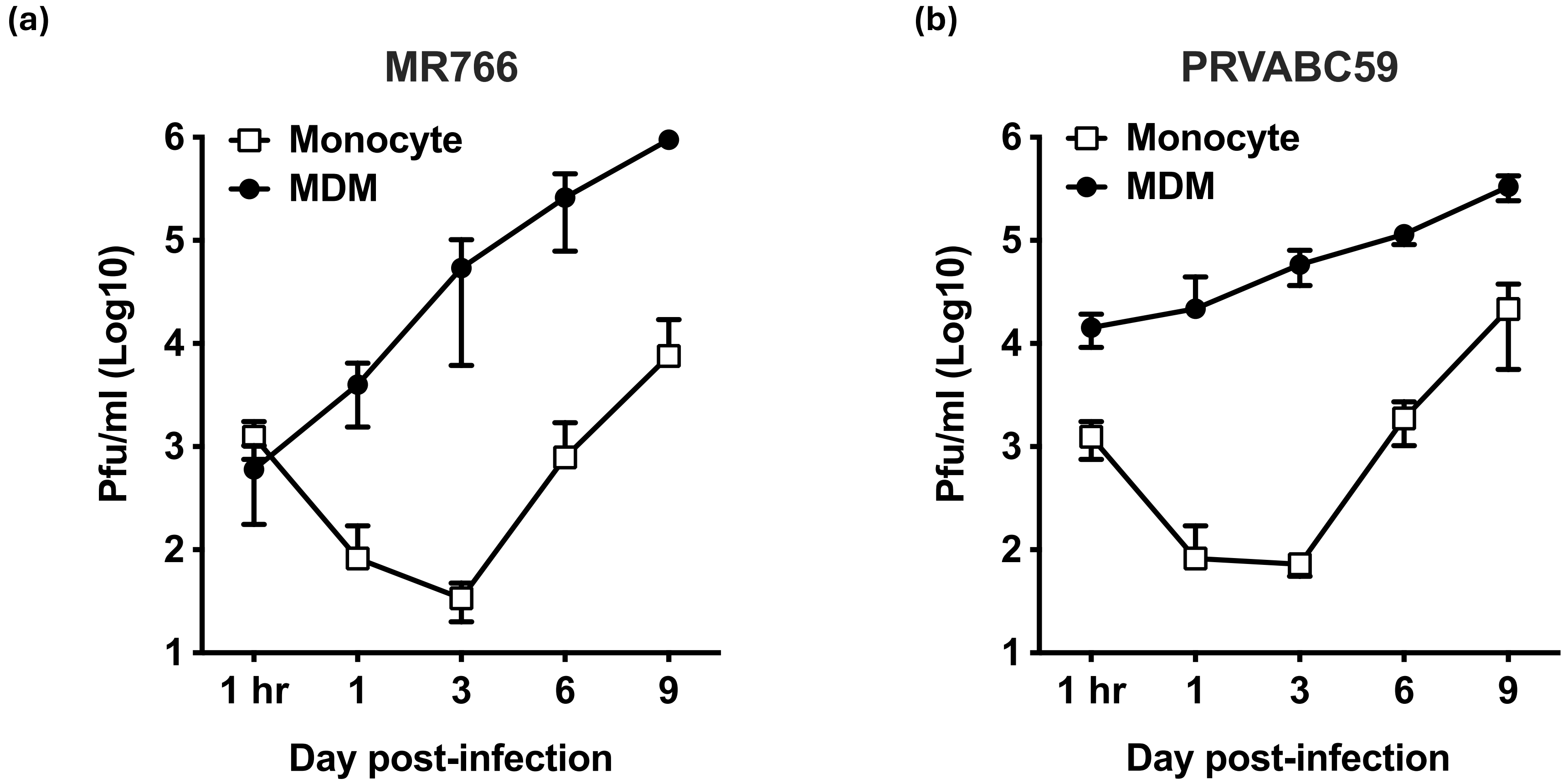

Legend: Superior replicative capacity of ZIKV (MR766 and PRVABC59 strains) in MDM vs. monocytes isolated from the same donor.

Supplemental Figure S2

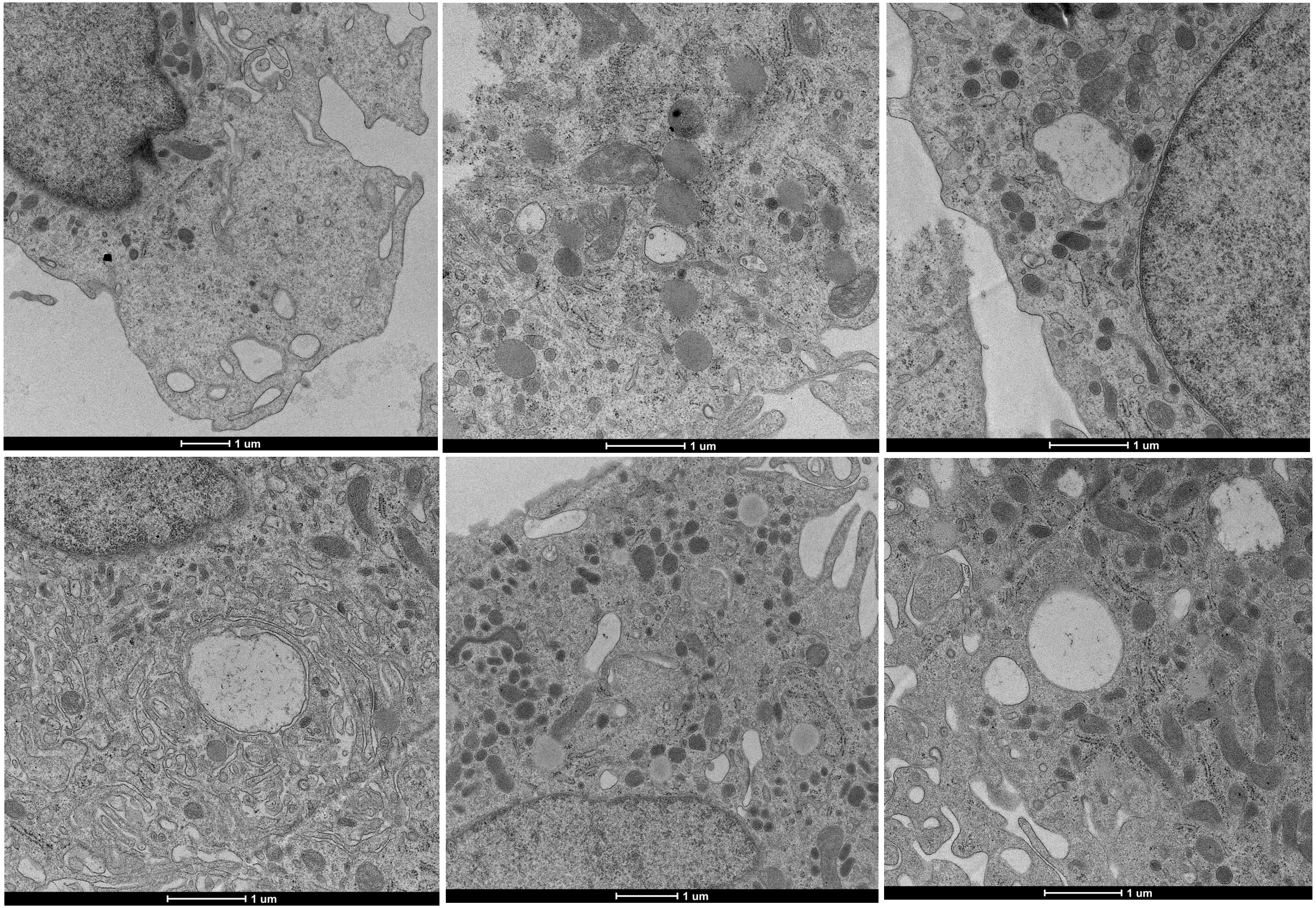

Legend: Close-up of empty vesicles in uninfected MDM

Supplemental Figure S3

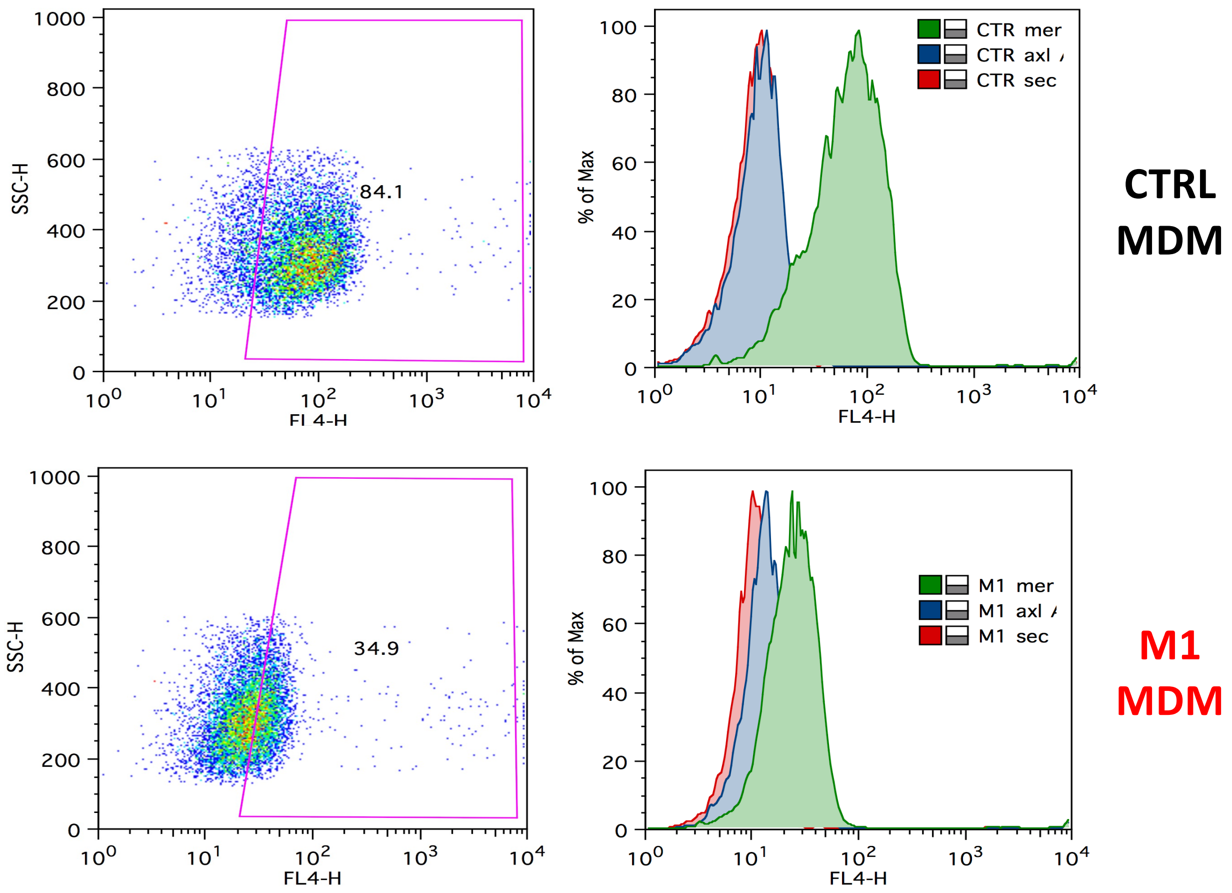

Legend: Downregulation of MERTK (mer) expression in M1-MDM; AXL (axl) expression was barely detectable over background (sec: secondary Ab) by FACS analysis.
